# Beyond traditional frontiers: robotic total pelvic exenteration

**DOI:** 10.1590/S1677-5538.IBJU.2019.0302

**Published:** 2020-09-02

**Authors:** Ashwin Sunil Tamhankar, Harit Chaturvedi, Gagan Gautam

**Affiliations:** 1 Department of Urooncology Tata Memorial Hospital Mumbai India Department of Urooncology, Tata Memorial Hospital, Mumbai, India;; 2 Max Institute of Cancer Care New Delhi India Max Institute of Cancer Care, New Delhi, India

## Abstract

**Introduction:**

Total pelvic exenteration with permanent fecal and urinary diversion is a rare, extensive and morbid surgical procedure reserved for locally advanced soft tissue tumors arising in the pelvis. A robot assisted approach with intracorporeal diversion has the potential advantage of decreasing the morbidity of this procedure, but has not been well described in literature.

**Materials and Methods:**

Using a da Vinci Xi® system, robot assisted total pelvic exenteration with intracorporeal diversion was performed in a 49 year old gentleman with a 13.1 x 9.6cm soft tissue sarcoma in pelvis. The salient steps involved sigmoid colon transection after high ligation of inferior mesenteric artery, control of posterolateral pedicles, opening of endopelvic fascia, apical dissection of urethra and completion of posterior dissection over presacral fascia to extract the specimen through a simultaneous perineal approach, extended pelvic lymphadenectomy and intracorporeal ileal conduit creation.

**Results:**

Console time, blood loss and length of stay were 410 minutes, 400cc and 9 days respectively. He had a minor complication in the form of lymphorrhea from perineal wound which resolved on Foley drain placement per urethra. Histopathology revealed epithelioid leiomyosarcoma with muscle invasion in bladder and rectum, resected with negative margins (pT2N0R0). All 32 lymph nodes were negative for metastases.

**Conclusion:**

Robotic approach to total pelvic exenteration is safe, feasible and replicates the principles of open oncological surgery while carrying the potential of decreasing the morbidity of this otherwise extensive surgery. This procedure is greatly facilitated by a thorough preoperative treatment planning by a multidisciplinary team.

